# A mobile NMR lab for leaf phenotyping in the field

**DOI:** 10.1186/s13007-017-0203-5

**Published:** 2017-06-28

**Authors:** Maja Musse, Laurent Leport, Mireille Cambert, William Debrandt, Clément Sorin, Alain Bouchereau, François Mariette

**Affiliations:** 1 IRSTEA, OPAALE, 17, avenue de Cucillé, 35044 Rennes Cedex, France; 2 Université Bretagne Loire, Rennes, France; 3 INRA, UMR 1349 IGEPP-Institut de Génétique, Environnement et Protection des Plantes, UMR INRA – Agrocampus Ouest-Université de Rennes 1, Domaine de la Motte, 35653 Le Rheu Cedex, France

**Keywords:** NMR relaxometry, Transverse relaxation (T_2_), Leaf senescence, Oilseed rape

## Abstract

**Background:**

Low field NMR has been used to investigate water status in various plant tissues. In plants grown in controlled conditions, the method was shown to be able to monitor leaf development as it could detect slight variations in senescence associated with structural modifications in leaf tissues. The aim of the present study was to demonstrate the potential of NMR to provide robust indicators of the leaf development stage in plants grown in the field, where leaves may develop less evenly due to environmental fluctuations. The study was largely motivated by the need to extend phenotyping investigations from laboratory experiments to plants in their natural environment.

**Methods:**

The mobile NMR laboratory was developed, enabling characterization of oilseed rape leaves throughout the canopy without uprooting the plant. The measurements made on the leaves of plants grown and analyzed in the field were compared to the measurements on plants grown in controlled conditions and analyzed in the laboratory.

**Results:**

The approach demonstrated the potential of the method to assess the physiological status of leaves of plants in their natural environment. Comparing changes in the patterns of NMR signal evolution in plants grown under well-controlled laboratory conditions and in plants grown in the field shows that NMR is an appropriate method to detect structural modifications in leaf tissues during senescence progress despite plant heterogeneity in natural conditions. Moreover, the specific effects of the environmental factors on the structural modifications were revealed.

**Conclusion:**

The present study is an important step toward the selection of genotypes with high tolerance to water or nitrogen depletion that will be enabled by further field applications of the method.

## Background

In the context of the increasing world population and the move towards more sustainable development, there is a need to increase agricultural productivity and to reduce the ecological footprint of plant production. To achieve these aims, genotypes need to be selected that can better adapt to environmental stresses using water and nutrients applied to the soil more efficiently, so plants can be grown with limited inputs. Large-scale phenotyping has been developed to assist such selection, as it allows characterization of plant adaptative traits in different agricultural systems. Studies have been conducted on a large number of plants with, for example, bulk methods of canopy spectral reflectance and absorbance [1]. On the other hand, to better understand plant functioning and adaptation to environmental changes, fine analyses have been conducted at organ and individual plant scales in a strictly controlled environment [2].

Plant response to the environment may differ in controlled and field conditions because of soil-climate and canopy architecture variability [3]. There is therefore a need to establish a link between measurements made in controlled conditions and field data. Currently, the trend is the development of new tools for fine phenotyping in the natural environment of the plant combined with large scale bulk methods. For example, non-destructive assessment of leaf chlorophyll by Multiplex [4] and Dualex [5] have been used for outdoor characterization of whole plant N status and leaf development. On the other hand, the use of leaf ranking or leaf ageing as indicators of developmental status is not relevant for genotype comparison when environmental conditions (light, temperature, wind, canopy structure, etc.) are responsible for marked heterogeneity among individual plants. The main problem with the methods cited above, and with more classical approaches like gas exchange measurements [6], is that they do not detect small differences in physiological traits between successive leaf ranks throughout the canopy.

The potential of nuclear magnetic resonance (NMR) relaxometry to finely evaluate the cell and tissue structure of oilseed rape leaves was recently demonstrated [7–9]. The NMR transverse relaxation time (T_2_), which is particularly sensitive to variations in water properties in plant tissues, was used to study changes in cell water status and distribution. As demonstrated in different plant tissues including leaves, differences in the physical and chemical properties of water in different compartments and the relatively slow diffusion exchange of water molecules between compartments are reflected by the multi-exponential relaxation times [9, 10]. Applied to a wide panel of leaves collected from oilseed rape plants of different genotypes grown in controlled conditions, NMR relaxometry was shown to be able to detect slight variations in senescence associated structural modifications in leaf tissues [7–9]. This characterization of the internal structure of the leaf allows accurate determination of leaf development stage independently of its position along the plant. In the context of fine phenotyping at individual plant scale in field conditions, the ability of NMR to identify leaves at the equivalent developmental stage and hence to allow plant traits to be compared is of particular interest. Moreover, NMR makes it possible to monitor changes in water exchanges and structural changes associated with remobilization efficiency [7–9].

Until now, the great majority of NMR and magnetic resonance imaging (MRI) studies on plants have been performed under controlled conditions in the laboratory. Current trends in the further development of the NMR/MRI method, largely motivated by phenotyping needs, are to extend investigations to in situ experiments (climate chambers, greenhouses or the natural environment) rather than to transport plants to the laboratory where the equipment is located. Relatively recent important technological advancements have been reported in mobile NMR devices. A single-sided open NMR sensor equipped with a permanent magnet for near surface studies, which allows free access to large objects, known as NMR MOUSE^®^ [11], was developed for a range of applications [12, 13], such as the characterization of soils, mortars and painting, as a logging tool for the petroleum industry, etc. It has also been used to study leaf water status in situ [14] and to determine the moisture fraction in wood [15]. Another approach has been to design specific NMR and/or MRI devices that can easily been placed or transported into climate chamber, greenhouse or in the field. For example, a small device known as NMR-CUFF [16] with a modified Halbach-type magnet that can be opened for sample positioning was developed by Windt et al. [16] and used to measure sap flow and the amount of water in plants in a climate chamber [17]. Another example of such designed instruments is the NMR system developed by Van As et al. [18] equipped with a permanent U-shaped magnet, with an access space of 2 cm used to study water content and transport in plants in greenhouses and climate chambers [19, 20]. Recently, a size-adjustable radiofrequency coil allowing investigation of plant samples of different diameters in a Halbach magnet has been proposed [21]. Finally, a few portable MRI systems have recently been developed mostly for the imaging of relatively small living trees for use in greenhouses [17, 22–24].

Only a limited number of NMR/MRI studies have been performed on plants in their natural environment. Capitani et al. used NMR relaxometry to investigate the water status of rockrose and holm oak leaves growing in sand dunes [14]. Okada et al. [25] reported the first outdoor MRI imaging of a living tree using a 0.3 T permanent magnet. A few years later, a permanent magnet equipped with flexible rotation and translation mechanism and combined with a mobile lift was used for outdoor imaging of pear tree branches up to 2 cm in diameter and up to 160 cm above ground level [26]. Jones et al. [27] designed a transportable MRI system offering an access space of 21 cm diameter for the imaging of living trees in the forest. Geya et al. [28] built a mobile MRI system with a 16 cm gap 0.2 T permanent magnet for measurements of the relaxation times and apparent diffusion coefficients of pear fruits in an orchard. This MRI system was recently shown to be able to measure water transport in trees outdoors [29].

The development of outdoor NMR and MRI measurements has faced two major challenges. The first concerns the NMR/MRI device itself and the effects of the environmental conditions on it. The system needs to be portable and easy to handle in different conditions. Further, the temperature drift of the magnet, the lack of homogeneity of the magnetic field and the variations in sample temperature can make it difficult to distinguish variations in the signal due to the biological changes under study from variations due to the system or measurement conditions. Some applications require accurate measurements and exploitation of the complete NMR signal. For example, in the specific case of characterization of the progress of senescence in oilseed rape leaves based on slight variations in multi-exponential relaxation parameters [8, 9], small variations in sample or magnet temperature can alter the results. Given the amplitude of possible variations in temperature due to weather conditions in the field, it is clear that the temperature of both the magnet and the sample have to be controlled. Furthermore, the inhomogeneous field, like that of the unilateral portable NMR system, is an additional source of relaxation, which shortens the T_2_ values measured [14]. The second challenge facing the study of plants grown in field conditions is related to the biological variability of the plant material. There are two potential sources of random variations in plant tissue characteristics that can alter the NMR signal. The main source is associated with the abiotic and biotic factors experienced by the plant throughout its development that lead to hardening of the leaf tissues. Further, compared to control conditions, plants grown in the field present higher variability in their canopy architecture, which is responsible for additional micro-environmental differences immediately prior to sampling. These aspects can cause erroneous results if the NMR measurements are simultaneously polluted by instabilities of the NMR device. The solution is to perform NMR measurements in the field using an NMR device that has been carefully checked in controlled laboratory conditions and to ensure the same measurement accuracy. Moreover, if field measurements are compared with those obtained on plants grown under well-controlled conditions, it is possible to identify the specific effects of variabilities caused by environmental factors.

The objective of this study was to demonstrate the potential of NMR to access information about the status and sub-cellular distribution of water in leaves from plants grown in natural conditions, thereby providing robust indicators of the stage of development directly in the field. To this end, a mobile NMR lab was designed for in situ measurements of the relaxation times of leaves from plants in their natural environment. A commercially available NMR spectrometer similar to that previously used for investigations of oilseed rape leaves [7–9] was used to create a mobile laboratory with the same performance as in the well-controlled laboratory experiments. The device was designed to be positioned at the edge of individual parcels in a field trial. The measurements made on the leaves of plants grown and analyzed in the field were compared to the measurements on plants grown in controlled conditions and analyzed in the laboratory. Results of the comparison showed that NMR can detect structural modifications in leaf tissues associated with senescence progress despite plant heterogeneity found under natural conditions.

## Methods

### NMR relaxometry

#### Instrumental setup

**Fig. 1:**
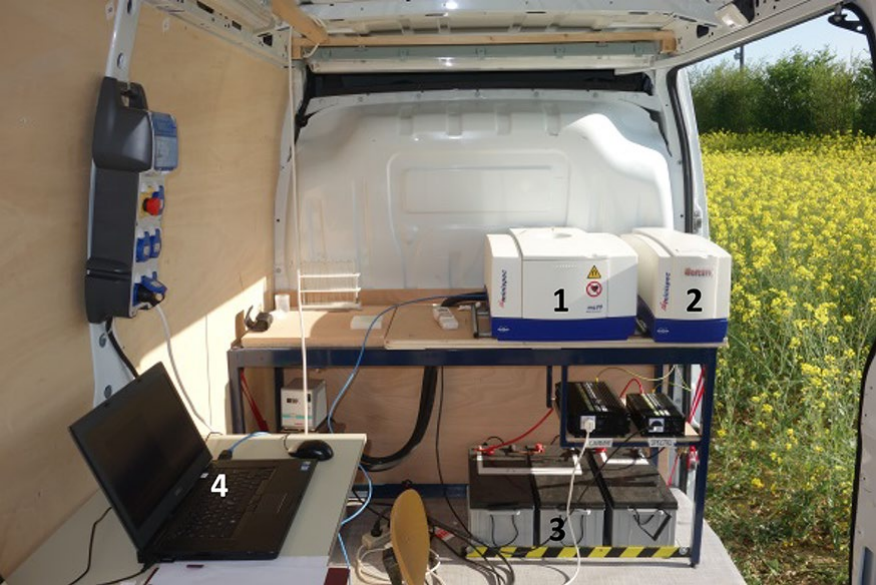
Mobile NMR laboratory with the NMR spectrometer including the magnet system and the probe assembly (*1*), the electronic control NMR unit (*2*), the battery (*3*) and a standard laptop computer for measurement control (*4*)

**Fig. 2:**
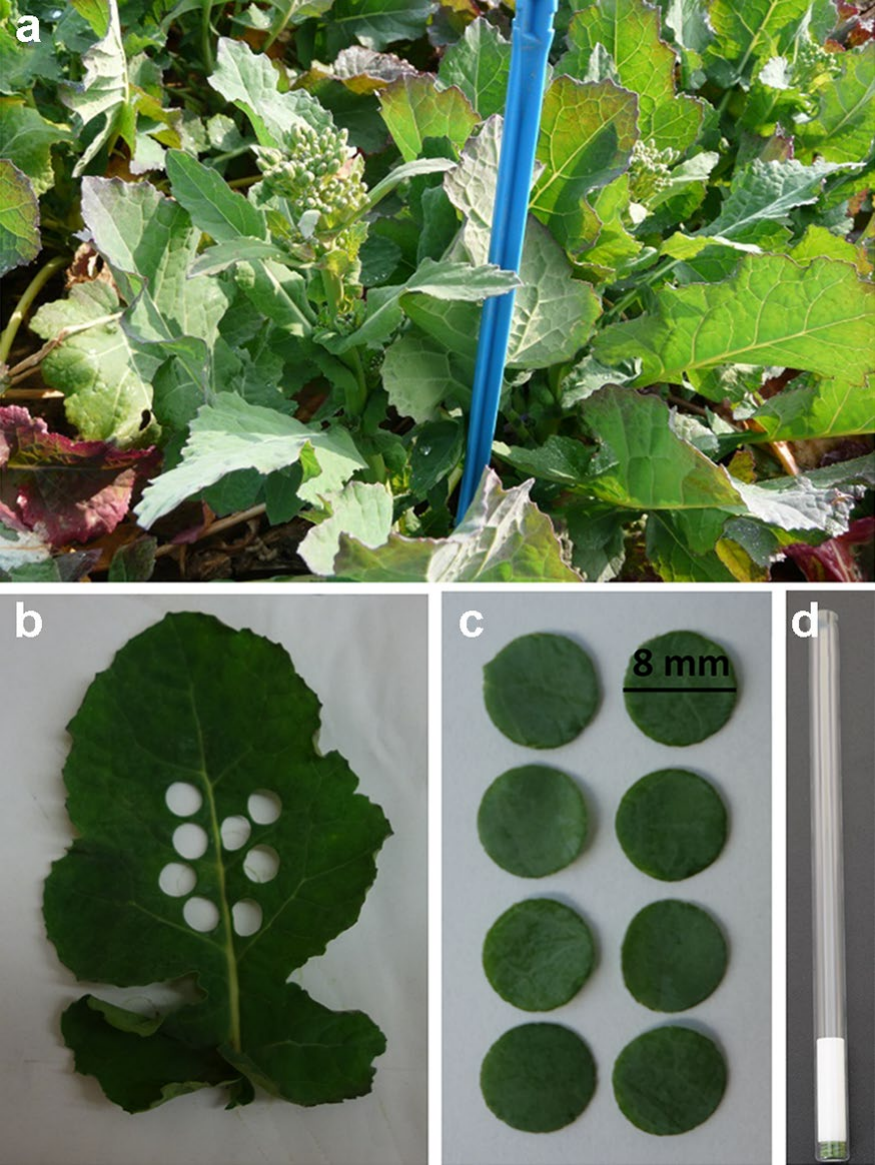
Oilseed rape plants at the end of stem elongation stage (**a**), leaf (LR −9) after disc sampling (**b**), eight discs cut from the leaf for NMR measurement (**c**) and NMR tube containing 8 discs, closed with a 2-cm long Teflon cap (**d**)

Transverse relaxation measurements were performed using a mobile NMR lab specifically set up for this purpose (Fig. 1). A commercially available 20 MHz spectrometer (Minispec PC-120, Bruker, Karlsruhe, Germany) equipped with a temperature control device connected to an optical fiber (Neoptix Inc, Canada) allowing ±0.1 °C temperature regulation was placed inside a van. The experimental device was powered by a battery. Such equipped van was positioned in the field close to the plants under investigation. No special care was taken to control the temperature inside the van. The leaf under investigation was cut from the plant (Fig. 2a) without uprooting the plant and, if the leaf was wet, wiped gently. Eight discs 8 mm in diameter were cut from each leaf of the plant studied (Figs. 2b, c). To obtain homogeneous tissues, the discs were cut in the middle of the limb on each side of the central vein and avoiding lateral second order veins. The discs were then placed in NMR tubes which were closed with a 2-cm long Teflon cap to avoid water loss during measurements (Fig. 2d). The temperature of the samples inside NMR probe was set at 18 °C.

#### Transverse relaxation signal acquisition and analysis

The transverse relaxation times was measured using the Carr–Purcell–Meiboom–Gill (CPMG) sequence. The signal was acquired with the 90°–180° pulse spacing of 0.2 ms. Data were averaged over 64 acquisitions. The number of successive echoes recorded was adjusted for each sample according to its T_2_. The recycle delay for each sample was adjusted after measurement of the longitudinal relaxation time (T_1_) with a fast-saturation-recovery sequence. The measurement time for T_2_ (including spectrometer adjustments and T_1_ measurement) was about 10 min per sample.

The CPMG signal was fitted using Scilab software according to the maximum entropy method (MEM) [30], which provides a continuous distribution of relaxation time components with no assumption concerning their number. In this representation, the peaks of the distribution are centered at the corresponding most probable T_2_ values, while peak areas correspond to the intensity of the T_2_ components. Signal intensity was expressed through the specific leaf water weight of the i^th^ signal component (LWW) expressed in g m^−2^. LWW of each CPMG component was calculated according to the equation:

1 $$ LWW_{i} = \frac{{I_{0Ri} \, \times \,m_{w} }}{A} $$

where I_0Ri_ is the relative intensity of the ith signal component expressed as a percentage of the total CPMG signal intensity, m_w_ is the water mass of the leaf discs used for NMR (in g) and A is the leaf disc area (in m^2^).

### Plant material

Oilseed rape (*Brassica napus* L., genotype Aviso) plants were grown in a field trial in Le Rheu, France (La Gruche, 48°8′17″N–1°48′11″O) during the 2014–2015 cropping season. The seeds were sown on the 10th of September, 2014 in plots measuring 6.75 m^2^ (4.5 × 1.5 m, 4 rows) at a density of 45 seeds m^−2^ and were grown under an optimal N regime. Four plots were used for this experiment corresponding to four repetitions. Measurements were made between the 23rd and the 26th of March 2015, at the end of the stem elongation stage (Fig. 2a), while floral buds were still closed (BBCH 55), on all fully expanded leaves (about 17 leaf ranks) of one individual plant (in four different plots, corresponding to four replicates).

### Indicators of leaf physiological status

#### Chlorophyll content

Before sampling the leaf discs for the NMR experiment, relative chlorophyll content per unit leaf area was estimated using a non-destructive chlorophyll meter (SPAD, Soil Plant Analysis Development; Minolta, model SPAD-502). The chlorophyll content of each leaf was estimated as the average of six independent measurements.

#### Water content

Leaf discs were kept in the closed NMR tubes until the end of the NMR experiment each day. The samples were transferred in the laboratory and water content was then determined by weighing before (fresh weight) and after drying (dry weight) in an oven at 70 °C for 48 h. Water content is expressed as a percentage of fresh weight.

### Comparison of the outdoor and laboratory NMR measurements

The outdoor NMR measurements made in the present study were compared with the NMR measurements performed on plants grown under controlled conditions and analyzed in the laboratory (data from [7, 8]). The objective of this comparison was to evaluate the NMR parameters as indicators of the leaf development stage in plants grown and analyzed in outdoor conditions. Data obtained on leaves from two different sets of plant were used for the comparison:

1. 32 non-vernalized oilseed rape plants of Tenor genotype, details are reported in [8]
2. 20 vernalized oilseed rape plants of Aviso genotype, details are reported in [7]

Like in the outdoor experiment, the NMR device used for transverse relaxation times in controlled conditions was a 20 MHz spectrometer (Minispec PC-120, Bruker, Karlsruhe, Germany). The CPMG measurements were performed at 18 °C with the 90°–180° pulse spacing of 0.1 ms and 64 signal averages.

Both NMR results and parameters describing physiological status measured were represented according to the NMR split scale. It was previously shown [8] that it is possible to use the split of the longest T_2_ signal component measured in the mature leaves to target leaves at the same developmental stage. In this representation, leaf rank zero is assigned to the leaf of the last leaf rank in which the split occurred, the subsequent leaf rank is numbered 1, etc. According to this scale, the older the leaf, the higher its rank, while negative ranks represent young leaves in which split has not yet occurred. This NMR split scale makes it possible to average values from data obtained in leaves located at different positions in the canopy. The NMR split scale is used in all the following figures.

## Results

### NMR field measurements

**Fig. 3:**
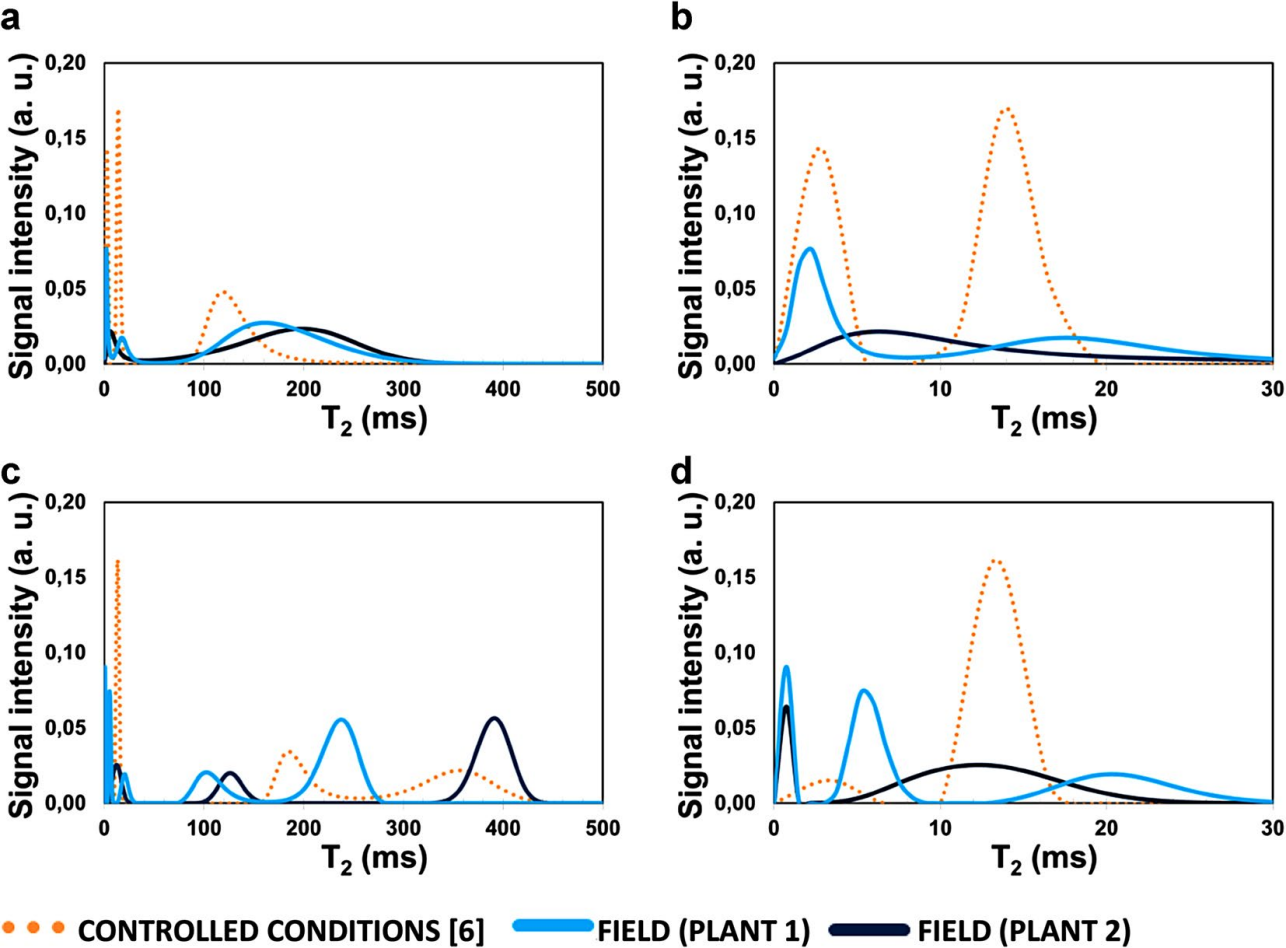
Distribution of transverse relaxation time (T_2_) calculated from the CPMG data for different leaves from plants grown in the field compared with the results obtained under controlled conditions from [7] Sorin et al., Botanical Studies 2016, 57; acknowledgment to Springer. **a** corresponds to the leaf rank −2 with **b** the zoom of the T_2_ distribution up to 30 ms. **c** corresponds to the leaf rank 0 with **d** the zoom of the T_2_ distribution up to 30 ms

Two typical examples (plants 1 and 2) of the transverse relaxation time distribution for two leaf development stages (leaf ranks −2 and 0) are shown in Fig. 3. For each stage, the curves are compared with the representative transverse relaxation spectrum obtained on leaves from plants grown under controlled conditions [7] at the same developmental stage according to the split scale. For the youngest leaves (leaf rank −2) from the field experiment, the longest T_2_ component corresponding to the vacuolar water was centered at 150–200 ms, which correspond to slightly higher values than those measured in the controlled conditions (Fig. 3a). This component corresponded to the largest amount of leaf water as it was observed in the plants grown under controlled conditions. For the older leaves (leaf rank 0), the vacuolar signal split into two components. The T_2_ values of the longest T_2_ components of the two spectra depicted in Fig. 3c were very different, illustrating the high variability of this parameter in senescing leaves, as already reported in [8, 9] and attributed to the high rate of structural changes responsible for the variations in the signal.

In addition to the vacuolar component described above, under controlled conditions, two other CPMG components were systematically observed. Figure 3b, d are zooms up to 30 ms on the T_2_ spectra shown in (a) and (c), respectively. The first one was centered at a few ms and represented a small percent of the water amount. It was always observed in the youngest leaves (leaf rank −2), disappeared with leaf age and was consequently observed only in some leaves of leaf rank 0. The second component was centered at about 15 ms and represented less than 20% of the water amount for all leaf ranks. Under field conditions, the signal differed from what was expected according to these results. Actually, the number of components detected in the T_2_ range 0–30 ms varied between one and three. In the case of two components (more than 50% of the leaves analyzed), the same general frame was found as that observed under controlled conditions (Fig. 3b leaf rank −2, plant 1 and d leaf rank 0 plant 2). In the case of three components detected (about 10% of the leaves), the additional component was detected with an intermediate T_2_ value of 5–8 ms (Fig. 3b leaf rank 0, plant 1). Finally, in the case of one component detected, the T_2_ value of this component was centered at 5–8 ms. Note that in all cases, the sum of the intensities of all these components represented approximately the same percentage of the total signal for a given leaf rank.

**Fig. 4:**
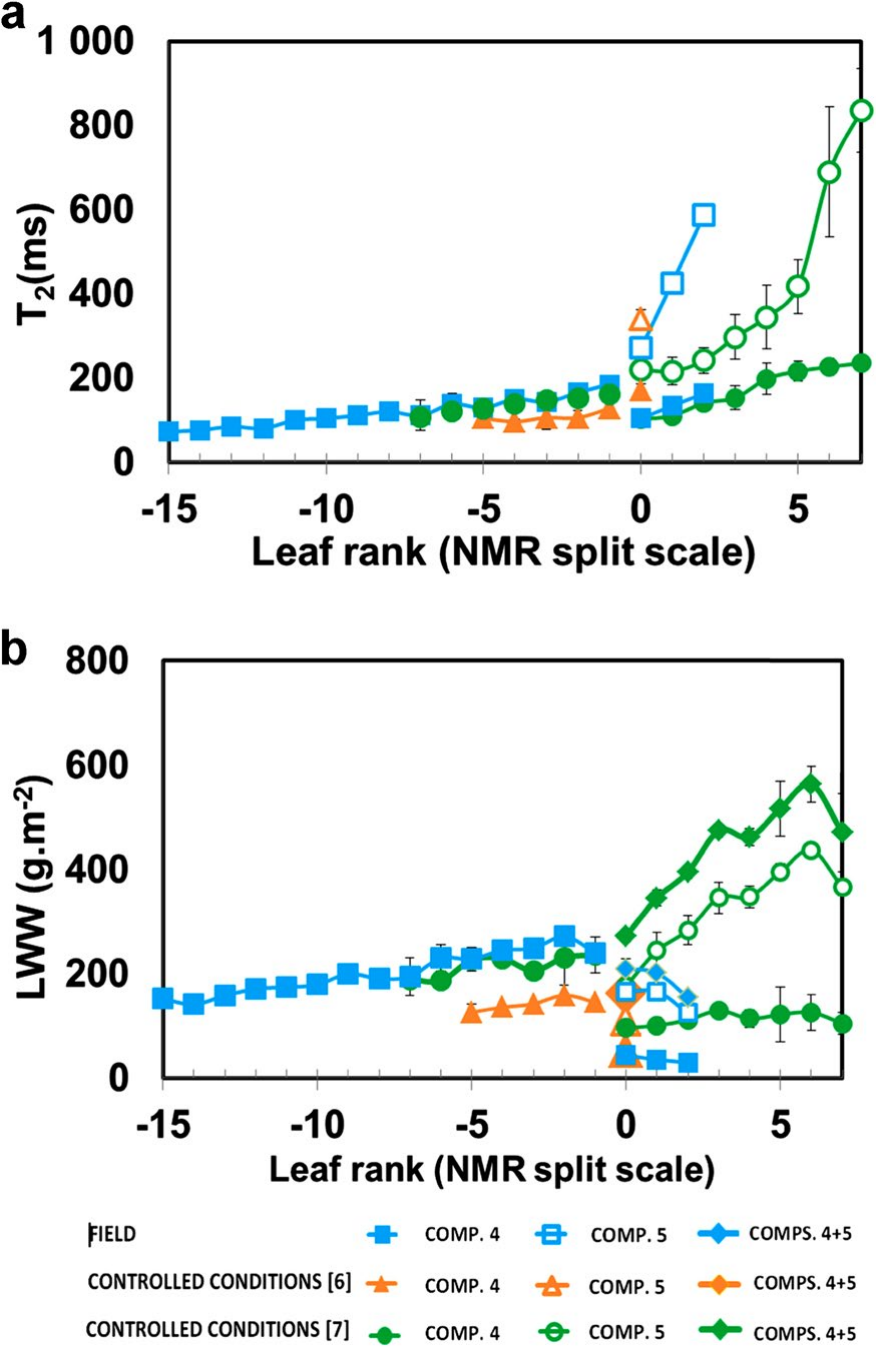
Transverse relaxation time (**a**) and specific leaf water weight (**b**) corresponding to vacuolar water of leaves during leaf development according to the NMR split scale leaf rank. The results of the field experiment are compared with the results obtained under controlled conditions [7]—Sorin et al., Botanical Studies 2016, 57; acknowledgment to Springer; [8]—Sorin et al., Planta. 2015, 24; copyright Springer. As the leaves were rearranged according to the NMR split scale after sampling, in the case of the field experiment the values corresponding to the leaf ranks between 0 and −10 are the averages ± standard deviations of data collected from leaves of four plants, for leaf rank −11 the data is the average ± standard deviations of data collected from leaves of three plants. For leaf ranks between −15 and −12 and between 3 and 1 less than tree measurements were available for analysis

Figure 4 shows the T_2_ (a) and LWW (b) values of the NMR signal components (4 and 5) associated with the vacuole during leaf ageing in plants grown in the field. Data from plants grown in controlled environment conditions extracted from [7, 8] are shown on the same graphs for the purpose of comparison. All the data are plotted according to the NMR split scale leaf rank (see “Methods”). In the case of the component 4, T_2_ values were reproducible over the whole leaf rank scale in all three growing conditions (Fig. 4a). After leaf rank 0, the maximum rate of the increase in T_2_(5) was the same in the different experiments (about 200 ms per leaf rank). However, while this maximum rate followed the appearance of the fifth component in plants grown in field conditions, it was delated for leaves from [8] where T_2_(5) increased at the maximum rate between leaf ranks +5 and +7.

Note that in the case of the field measurements, it was possible to analyze leaves characterized by leaf ranks up to −15, corresponding to very young leaves. This made it possible to confirm the increasing trend in the value of T_2_(4) with ageing of the leaf. Considering differences in genotypes, vernalization and environmental factors between plant growing conditions, Fig. 4a shows that it was possible to establish a master curve describing the structural changes in young leaves (negative leaf ranks). After LR = 0, T_2_ values describing structural leaf changes appeared to be more affected by environmental conditions.

According to [7–9], the NMR intensities of each signal component (Fig. 4b) are expressed in leaf water weight (Eq. 1). Like in the plants grown under controlled conditions, LWW (4) increased steadily until leaf rank 0, reflecting the progressive increase in the amount of vacuolar water with aging. An unexpected result was that for the positive leaf ranks associated with the four oldest leaves, LWW (4 + 5) corresponding to vacuolar water decreased in the field experiment in contrast to the data reported in [7, 8].

### Physiological characterization of leaf development

**Fig. 5:**
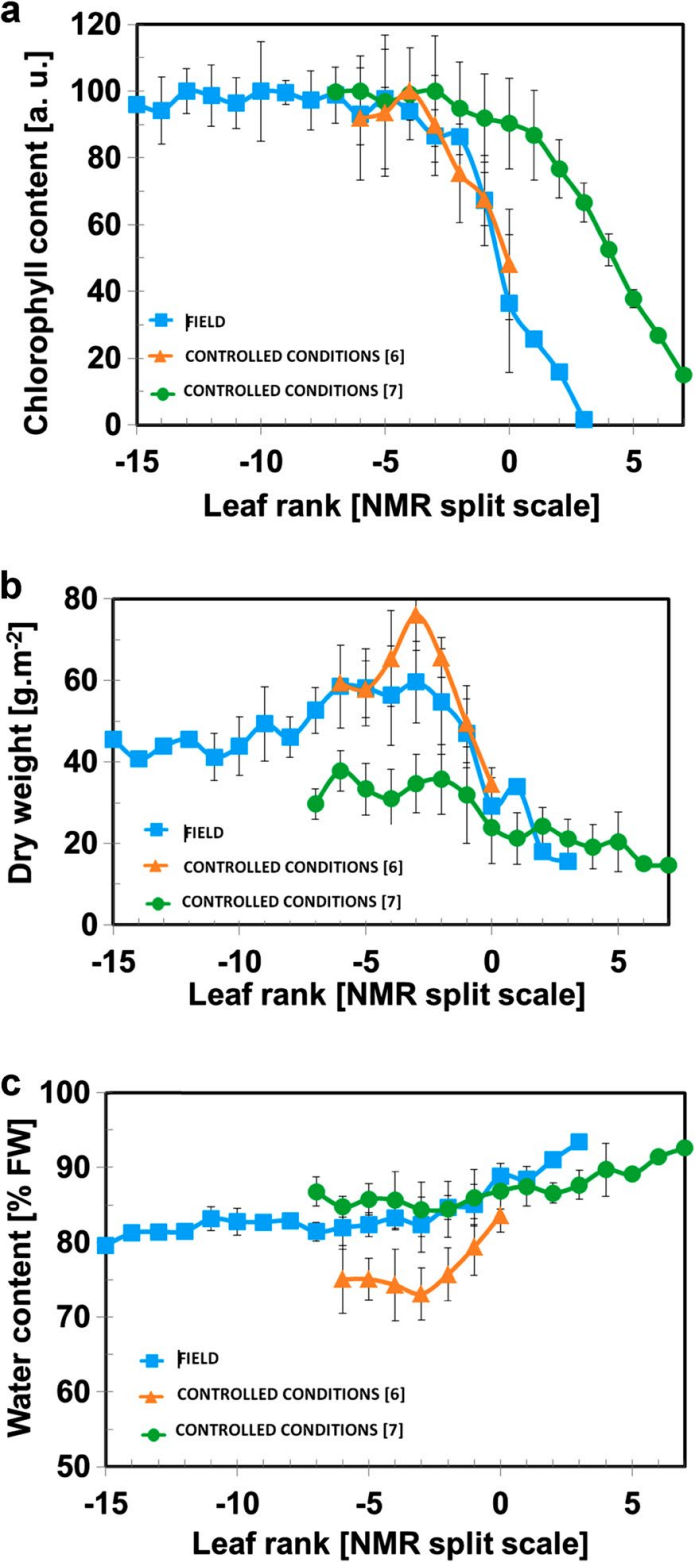
Changes in chlorophyll content (**a**), dry weight (**b**) and water content (**c**) during leaf development according to the NMR split scale. The results of the field experiment are compared with the results from obtained under controlled conditions [7]—Sorin et al., Botanical Studies 2016, 57; acknowledgment to Springer; [8]—Sorin et al., Planta. 2015, 24; copyright Springer. As the leaves were rearranged according to the NMR split scale after sampling, in the case of the field experiment the values corresponding to the leaf ranks between 0 and −10 are the averages ± standard deviations of data collected from leaves of four plants, for leaf rank −11 the data is the average ± standard deviations of data collected from leaves of three plants. For leaf ranks between −15 and −12 and between 3 and 1 less than tree measurements were available for analysis

Changes in physiological traits during leaf development were described through general parameters (Fig. 5) i.e. chlorophyll content, dry weight and water content. These data, like those presented in Fig. 4 are presented according to the NMR split scale and compared with data obtained on plants grown in controlled environment conditions extracted from [7, 8]. The chlorophyll content measured in the field was at its maximum value from leaf rank −13 to −4 and decreased markedly from leaf rank −2 (Fig. 5a) to a very low value for the oldest leaves. These results show that our study was performed on a large panel representing a relatively wide range of young, mature and senescent leaves. In controlled conditions, the same general trend was observed. However, the curve representing chlorophyll content measured on non-vernalized plants [8] started to decrease for LR 1, which corresponded to older leaves, compared to the curves obtained on vernalized plants independently of the trial. In field conditions, the dry weight (Fig. 5b) increased slightly from leaf rank −15 to −2, reflecting the production of biomass associated with leaf growth. It then dropped from leaf rank −1, reflecting the loss of about 75% of leaf biomass explained by major remobilization at leaf senescence. This was consistent with the data obtained in controlled conditions [7, 8]. Note that in non-vernalized plants, specific dry weight had lower values than in vernalized plants. In field conditions, leaf water content increased slightly from the youngest leaves to leaf rank −3, indicating that leaf tissue was able to maintain cell homeostasis. From leaf rank −4, the loss of dry matter was more marked than that of water, resulting in an increase in water content. The same general trend was observed in controlled conditions, although the water content was systematically lower in the vernalized plants grown under controlled conditions.

## General discussion

### NMR as a phenotyping tool in field conditions

The results of the present study show that a mobile NMR lab makes it possible to successfully perform outdoor NMR measurements under controlled conditions usually obtained in the laboratory that ensure optimum measurement accuracy. Using this approach, it was possible to compare the NMR signal from plants grown under well-controlled conditions (growth cabinet) with the signal from the plants grown in the field.

The trend in the transverse relaxation times associated with vacuolar water revealing changes in leaf structure during senescence was very similar to that previously measured on plants grown under controlled conditions. This shows that, despite the great variability of the environmental factors during plant growth and throughout the canopy (marked variations in temperature and humidity, light exposure, wind, etc.), the NMR method can provide robust indicators of the leaf development stage of plants grown in the field. The results of the present outdoor study, confirmed previous data [7–9] showing that enlargement of palisade cells reflected by the signal split correspond to a rather late leaf senescence event in oilseed rape leaves. However, the present study using data collected from a wider range of leaf ranks also demonstrated that structural changes were initiated very early in mature leaves. Indeed, the physiological process at the origin of this late event (split of the NMR component corresponding to the vacuolar water) highlighted by a continuous increase in the T_2_ value of the longest component corresponding to vacuolar water appears to have been initiated while the chlorophyll and dry matter content were still high.

Within the common framework of changes in relaxation times, some differences in the NMR parameters measured on plants grown under different conditions were observed and are discussed in the following paragraph.

### Impact of the environmental conditions on leaf development

As mentioned above, some differences were observed in the NMR parameters measured on plants grown under different conditions (Fig. 4a, b). These differences revealed modifications in leaf development pattern caused by heterogeneous climate conditions with marked and abrupt changes in temperature and/or humidity. Although the T_2_ of the signal component associated with vacuolar water before the split (leaf rank 0) was very similar, a difference was observed in the leaf ranks at which the maximum rate of the increase in the T_2_ value occurred. This indicated that changes in the vacuolar volume associated with the T_2_(5) increase [8] were precocious in field plants. This is in accordance with the measurements of chlorophyll content (Fig. 5a) and is probably explained by more marked changes in environmental conditions during leaf development.

The environment perceived by plants has an effect on the progress of leaf senescence but not necessarily on the onset of senescence [31]. It is well known that several factors, including an unbalanced sink-source ratio, may initiate leaf senescence. For instance, in the case of a high source-sink ratio and photoassimilates, inhibition of feedback by photosynthesis may trigger senescence, whereas in opposite high sink activity may trigger leaf senescence through the remobilization of nutrients (mainly nitrogen) [32]. The effect of N status on the induction of senescence may also be driven by plant archichecture and the quantity and quality of light that reaches the leaf [33]. In sunflower, it has been reported that nitrogen export is promoted by a low red/infra-red ratio rather than by the amount of light [34]. Nevertheless, in the present study, it seems that the main discriminating factor at the origin of the differences observed in the senescence patterns described by NMR signal components corresponding to the vacuolar water and other parameters was the vernalization process and the physiological status of plants and not to the heterogeneous conditions prevailing in the field experiment (in contrast to the homogenous conditions in the growth cabinet). The role of vernalization in the regulation of leaf senescence has been reviewed in [35]. Cold temperatures can affect both plant development and leaf senescence [36]. Vernalization has an impact through different mechanisms. While infertility increases the life span of the whole plant through the production of additional young leaves [37], as was the case in the non-vernalized plants analyzed in [8], the vernalization process initiates the development of the new reproductive organ with high sink activity. It has also been reported that leaf senescence may speed up with flowering [38]. In the present study, measurements were made at the stem elongation stage during which the process of nutrient remobilization from the old leaves was emphasized. Finally, it should be noted that, in the experiment corresponding to the non-vernalized plants [8], the nutritive solution was supplied twice a week, while in the case of the vernalized plants ([7] and the present study) N was supplied according to the plant development. In both cases, the plants were grown under optimum nutrient conditions but in the case of the vernalized plants, the level of the available nutrients in the soil was irregular.

The young leaves from the vernalized plants (present experiment and [7]) were characterized by higher dry weight than those of the non-vernalized plants [8]. This is probably due to a more active cell division induced by cold stress [39] associated with a higher number of cell layers. However, the dry mass decreased markedly in senescing leaves sampled on vernalized plants, whereas the decrease was minor in leaves taken from non-vernalized plants. This phenomenon has been explained by the appearance of large intercellular grass-filled spaces and a resulting decrease in the number of cells [7]. This means that although senescence is characterized by an increase in vacuole volume [7–9] reflected by an increase in water content and in T_2_, the total amount of the vacuolar water expressed by LWW (4 + 5) decreased in senescing leaves.

Concerning the NMR signal components corresponding to water in cell compartments other than vacuoles (T_2_ range up to 30 ms), very similar results were obtained in the field experiment and in the experiments conducted under controlled conditions in about 50% of the leaves analyzed. In the remaining leaves, the number of components detected in this T_2_ range was one or three, instead of two components as expected from [7, 8]. Considering that in all cases, the sum of the intensities of all these components (one, two or three) represented appromimately the same percentage of the total signal for a given leaf rank, it can be assumed that these T_2_ peaks corresponded to the same water pools (apoplastic water and water inside starch granules and chlorophyll) as proposed in [8]. However, due to the heterogeneous environmental factors (for example, the amount of light received by the leaves), it was not possible to distinguish the corresponding water pools according to their T_2_ values in all cases. It will be interesting to clarify this point in further experiments, although this is not crucial for the purpose of phenotyping.

Considering a negative shift of five leaf ranks for the chlorophyll content curve (Fig. 5a) corresponding to the non-vernalized plants, a perfect match was observed in the three sets of data. It would be therefore possible to argue that structural changes associated with senescence occur earlier in non-vernalized plants. This can be explained by the fact that, as mentioned above, leaf tissues of non-vernalized plants had fewer cell layers and a thinner cuticle. For this reason, it is possible to conclude that (1) structural changes occurring in the leaf throughout the canopy are a constant feature in the development of the leaf in oilseed rape independently of environmental and growing conditions, (2) it is possible to monitor these structural changes with NMR relaxometry in the field as in a controlled environment, but (3) the NMR split scale should only be used for comparison within a set of measurements made in the same set of growing conditions.

### NMR experiment in outdoor conditions

Until now, only a few mobile NMR [14] and MRI [27–29] systems have been used to investigate plants in their natural (outdoor) environment. Although the constraints of NMR and MRI approaches are not exactly the same, both techniques have to deal with the effects of the environmental conditions on the NMR/MRI devices. All outdoor measurements were performed with permanent magnets that are the best suited for mobile devices but have the disadvantage of being very sensitive to variations in temperature. In the first instance, the temperature drift of the magnetic field is limited by thermal insulation of the magnet [27, 28] and is generally further compensated by using the field frequency lock approach. However, because changes in air temperature in outdoor environments are usually much larger than in standard laboratories, shifts in temperature can be a major problem in outdoor measurements [26]. Another problem with outdoor measurements is the role of the temperature variations in the NMR signal. In plant tissues, the dependence of the distribution of water proton transverse relaxation times on temperature is complex, as several contributions may overlap [40]. Indeed, the temperature affects molecular mobility and the diffusive exchange of water between the subcellular compartments (diffusion coefficients and membrane permeability). As a result, both T_2_ and peak amplitudes change with temperaure, and are thus a possible source of interpretation errors.

Malone et al. [24] isolated the variation in the signal due to biological factors from that due to changes in the temperature of the detector. These authors characterized the sensitivity of their system to temperature and built a model that accounted for the particular linear dependence of the detector circuitry on temperature for the prediction of the variation in the NMR signal. The method was shown to be effective in a greenhouse experiment in which the average daily temperature variation was 10 K, with an average daily high near 305 K. However it should be tested for higher temperature variations before the method is used in outdoor conditions. The temperature stability of the samples is also critical for measurements of relaxation times as both signal ampliture and relaxation times depend to a great extent on temperature. This issue has been addressed in only a limited number of studies. Windt et al. [17] addressed a problem of variations in the temperatures of the sample and of the spectrometer by continuously monitoring the temperature of the tree stem in the NMR device and the temperature of the spectrometer. They observed slight differences in the amplitude of the NMR signal, which they attributed to variations in the temperature of the object (due to a shift in the Boltzmann equilibrium) and temperature-induced differences in the signal amplification factor of the spectrometer. A temperature correction factor was applied to compensate for these differences. Another possible approach is to equip the NMR device with a temperature control device for a sample. Anferova et al. [41] developed two different types of mobile temperature control units compatible with the NMR-MOUSE for analysis of rubber by transverse NMR relaxation. For this application, stable temperatures of the samples were critical, and temperature stability of better than 0.5 °C was needed for good reproducibility of measurements. The use of the device developed and tested here extends the range of possible investigations with the NMR-MOUSE to samples in environments with marked variations in temperature.

In the present study, the effects of the environmental conditions on both the device and the sample were controlled. The operating temperature of the benchtop NMR device magnet was maintained through a magnet heating system. The stable temperature of the sample was ensured by a variable temperature control units within 0.1 °C. The NMR mobile laboratory therefore made it possible to perform measurements in exactly the same controlled conditions throughout the experiment, despite the fact the measurements were performed in an environment with no temperature regulation.

As described in “Methods”, after the NMR experiment, the samples were transferred to the laboratory for the determination of water content and dry weight of leaves. These measurements were not possible in the field, as weighing the samples requires a precision balance, which was shown to be sensitive to transport. However, by measuring the free induction decay (FID) signal in addition to the CPMG curve, it is possible to descriminate between water and dry matter and to use the relationship established in [9] to estimate the water content and dry mass of the leaf samples. This would make it possible to overcome the difficulty of using a balance in the field in further experiments.

## Conclusion

The mobile NMR laboratory developed in this study was shown to be able to perform accurate outdoor characterization of oilseed rape leaves throughout the canopy. The approach used enabled in situ assessment of physiological status of leaves from plants grown in their natural environment without disturbing the plants themselves. The study enabled the comparison between the patterns of NMR signal evolution from plants grown under well-controlled conditions and plants grown in the field. The method described here provides new opportunities for fine phenotyping and monitoring of plant development in the natural environment. It can be used for the selection of oilseed rape genotypes with high tolerance to water or nitrogen depletion. Future investigations should extend the method to other crops.
